# Book Notices

**Published:** 1869-09-15

**Authors:** 


### BOOK NOTICES.

### IMPORTANT MEDICAL WORKS.

CONTRIBUTIONS RELATING TO THE CAUSATION AND PREVENTION OF DISEASE, AND TO CAMP DISEASES; TOGETHER WITH A REPORT OF THE DISEASES, ETC., AMONG THE PRISONERS AT ANDERSONVILLE, GA. Edited by AUSTIN FLINT, M.D. In one volume 8vo. Cloth, extra, $6.50.

In this volume, Dr. Flint has brought together papers contributed by eminent medical men, from material gathered in the prosecution of the U. S. Sanitary Commission's labors. The titles of some of these papers are as follows:

On the various Influences affecting the Physical Endurance and the Power of Resisting Diseases of the Men composing the Volunteer Armies of the United States, by Prof. Robert Bartholow, M.D.;

On the Causation of Disease, etc., by A. J. Phelps, M.D.;

On Army Alimentation, by Sanford B. Hunt, M.D.;

On Vaccination, Revaccination and Spurious Vaccination, by Elisha Harris, M.D.;

On Scurvy, Diarrhœa and Dysentery, by Sanford B. Hunt, M.D.;

On the Diseases of Nerves, resulting from Injuries, by S. Weir Mitchell, M.D.

The third section of the volume is occupied with the official report of investigations upon the diseases of the Federal prisoners confined in Camp Sumpter, Andersonville, Ga., by Prof. Joseph Jones, M.D. Prof. Jones was a Confederate Surgeon during the war, and his investigations made at Andersonville were by direction of the Confederate authorities.

---

In addition to the above volume there have been published the following valuable works:

I. INVESTIGATIONS IN THE MILITARY AND ANTHROPOLOGICAL STATISTICS OF AMERICAN SOLDIERS. By BENJAMIN APTHORP GOULD, Ph. Dr., Actuary to the U. S. Sanitary Commission. In one volume 8vo. Cloth, $6.50.

The questions of nativity, age, stature, complexion, color of hair and eyes, occupations, dimensions of body, proportions of pody, dimensions and proportions of head, weight and strength, pulmonary capacity, respiration and pulse, vision, education—these and others embraced in them are illustrated and discussed by aid of a multitude of classified facts.

II. HISTORY OF THE UNITED STATES SANITARY COMMISSION, being the General Report of its work during the war of the rebellion. By CHARLES J. STILLE. In one volume 8vo. Cloth, extra, $3 50.

The inspection of Camps and Hospitals, Hospital Transport Service, Hospital Supplies, Relief on the Battle-field, Special Relief, Warfare against Scurvy, Scenes of Labor at Vicksburg, Chattanooga, Fredericksburg, Gettysburg, The Wilderness, Newberne—these and other divisions of his subject are all woven into an animated narrative, free from cumbersome detail, but fresh with illustration, possessing the great interest of a picturesque historic work.

Published and for sale by Hurd & Houghton, New York.

H. O. Houghton & Co., Riverside, Cambridge, Mass.

The volumes will be supplied by any bookseller.

Further notice of these volumes will be given soon. Meanwhile, we recommend their perusal to our readers.

